# Perspective: A Neuro-Hormonal Systems Approach to Understanding the Complexity of Cryptorchidism Susceptibility

**DOI:** 10.3389/fendo.2018.00401

**Published:** 2018-07-23

**Authors:** Julia S. Barthold, Richard Ivell

**Affiliations:** ^1^Nemours Biomedical Research, Division of Urology, Alfred I. duPont Hospital for Children, Wilmington, DE, United States; ^2^School of Biosciences and School of Veterinary Medicine and Science, University of Nottingham, Sutton Bonington, United Kingdom

**Keywords:** cryptorchidism, testicular descent, gubernaculum, androgen, insulin-like 3, systems biology

## Abstract

Nonsyndromic cryptorchidism is a common multifactorial, condition with long-term risks of subfertility and testicular cancer. Revealing the causes of cryptorchidism will likely improve prediction and prevention of adverse outcomes. Herein we provide our current perspective of cryptorchidism complexity in a synthesis of cumulative clinical and translational data generated by ourselves and others. From our recent comparison of genome-wide association study (GWAS) data of cryptorchidism with or without testicular germ cell tumor, we identified RBFOX family genes as candidate susceptibility loci. Notably, RBFOX proteins regulate production of calcitonin gene-related peptide (CGRP), a sensory neuropeptide linked to testicular descent in animal models. We also re-analyzed existing fetal testis transcriptome data from a rat model of inherited cryptorchidism (the LE/orl strain) for enrichment of Leydig cell progenitor genes. The majority are coordinately downregulated, consistent with known reduced testicular testosterone levels in the LE/orl fetus, and similarly suppressed in the gubernaculum. Using qRT-PCR, we found dysregulation of dorsal root ganglia (DRG) sensory transcripts ipsilateral to undescended testes. These data suggest that LE/orl cryptorchidism is associated with altered signaling in possibly related cell types in the testis and gubernaculum as well as DRG. Complementary rat and human studies thus lead us to propose a multi-level, integrated neuro-hormonal model of testicular descent. Variants in genes encoding RBFOX family proteins and/or their transcriptional targets combined with environmental exposures may disrupt this complex pathway to enhance cryptorchidism susceptibility. We believe that a systems approach is necessary to provide further insight into the causes and consequences of cryptorchidism.

The undescended testis has been the object of continued interest over many years. In April 2018, a search for “cryptorchidism” in PubMed (https://www.ncbi.nlm.nih.gov/pubmed) yielded nearly 10,000 articles spanning almost 100 years. Cryptorchidism has been an area of interest because of its inheritance patterns in domesticated mammals, its high prevalence in man (2–9% of all newborn boys) and its co-morbidities, including subfertility and testicular cancer. Despite sustained and focused attention, the pathogenesis of cryptorchidism and its associated conditions remain poorly understood. Indeed, the more we study the condition, the more complex it seems to become. It stands to reason that better knowledge of the global mechanisms of testicular descent would provide important insight into the causes of cryptorchidism, and would allow us better to predict and prevent this common birth defect and its consequences.

Through our work over the years studying testicular descent and cryptorchidism in animal models and in man, we now propose a consolidated model of testicular descent comprising multilevel integration of neuro-hormonal signaling, and that cryptorchidism results when a combination of genetic and environmental factors target this integrated pathway. Below, we present relevant published and unpublished evidence supporting this perspective.

## Leydig cell hormones work separately and together to bring about testicular descent

Enlargement and regression/migration of the gubernacular ligament (gubernaculum) connecting the ventral pole of the testis/epididymis to the body wall in the inguinal region are indispensable for testicular descent (1, 2). Based on Hutson's hormonal regulation model (3), transabdominal (Phase 1) and transinguinal (Phase 2) descent are distinct, and largely regulated by a non-androgenic hormone, now known to be insulin-like 3 (INSL3), and androgens, respectively. At the same time in most mammals a second ligament, the cranial suspensory ligament (CSL), linking the dorsal pole of the testis to the dorsal surface of the body cavity close to the embryonic kidney, needs to dissolve. Studies of androgen receptor knockout (ARKO) and *tfm* (testicular feminization) male mice, androgen-exposed female mice, and anti-androgen-exposed rats clearly show that the CSL is regulated by androgens and that its persistence leads to cryptorchidism (4–9). Yet CSL regression is thought to facilitate transabdominal rather than transinguinal, descent. Other inconsistencies exist, complicating efforts to show that distinct hormones regulate distinct phases of descent across species. Remodeling of the CSL may not truly occur, or may be less relevant in human fetuses (10, 11). Some human subjects with complete androgen insensitivity syndrome have testes located close to ovarian position (12). Studies in rodents suggest that INSL3 overexpression leads to partial ovarian descent and transinguinal migration of the processus vaginalis, leading to hernia (13), which could be interpreted as an evolutionary relic of a primitive mode of testis excorporation. Similarly, RXFP2, the INSL3 receptor, appears to augment the role of androgens in transinguinal descent (14) and together with AMH may influence gubernacular cell proliferation in culture (15).

Knockout experiments in mice clearly show independent requirements for INSL3/RXFP2 and androgens in testicular descent (16, 17); nevertheless causative mutations in *INSL3, RXFP2, AR* (androgen receptor) or the Leydig cell regulator *NR5A1* (steroidogenic factor-1), are rare in cases of cryptorchidism (18, 19). WNT signaling appears to be a downstream target of both INSL3 and androgen in the fetal rat gubernaculum (20, 21), and cryptorchidism and/or gubernacular maldevelopment occur in mice with transgenic deletion of WNT pathway genes, such as *Sfrp1/Sfrp2, Wnt5a, Ctnnb*, or *Vangl2* (16, 22–25). Yet none of these genes has been implicated in human cryptorchidism. If INSL3 and androgen are each indispensable for testicular descent, fetal Leydig cell function must play a central role in cryptorchidism susceptibility. This is strongly echoed by studies on the effects of maternal exposure to phthalates in rats where the fetal Leydig cells are seen as primary targets for this endocrine disruptor, leading to a reduction in both INSL3 and testosterone production as well as cryptorchidism (26). Yet the effects of phthalates appear to be species-specific, with humans and mice seemingly more resistant to these inhibitory effects on testicular hormone production (27, 28). While detailed studies of Leydig cell function during the prolonged process of testicular descent in human fetuses are unavailable, it is reasonable to assume that genetic and/or environmental factors that alter this function may contribute to cryptorchidism.

## The sensory neuropeptide calcitonin gene-related peptide (CGRP) plays a role in testicular descent

A role for CGRP in testicular descent and cryptorchidism is supported by *in vitro* and *in vivo* rat studies [reviewed in (29)]. Experiments in newborn rodents showed that transection of the genitofemoral nerve (GFN; which innervates the gubernaculum) causes cryptorchidism, and that CGRP release by the sensory limb of the GFN regulates proliferation and motility of the gubernaculum. Hutson and colleagues found evidence for interaction between CGRP and androgens in rodent models (30, 31), and in the absence of clear AR expression in the fetal gubernaculum (32, 33) they theorized that androgens indirectly modulate CGRP via effects on surrounding AR+ mammary tissue. However, other data suggest that the fetal gubernaculum does express its own functional AR (17, 21, 34–36). Clinical data have not shown an association of genetic variants in the CGRP pathway with cryptorchidism (37). However, we recently found a potential role for RBFOX proteins, which regulate production of CGRP, in genetic association analyses of cryptorchidism (see below), which may provide evidence supporting a role for CGRP in humans.

## Heritable cryptorchidism in the LE/orl rat is associated with multi-level dysregulation of testicular descent, and multilocus inheritance of cryptorchidism

**Figure 1:**
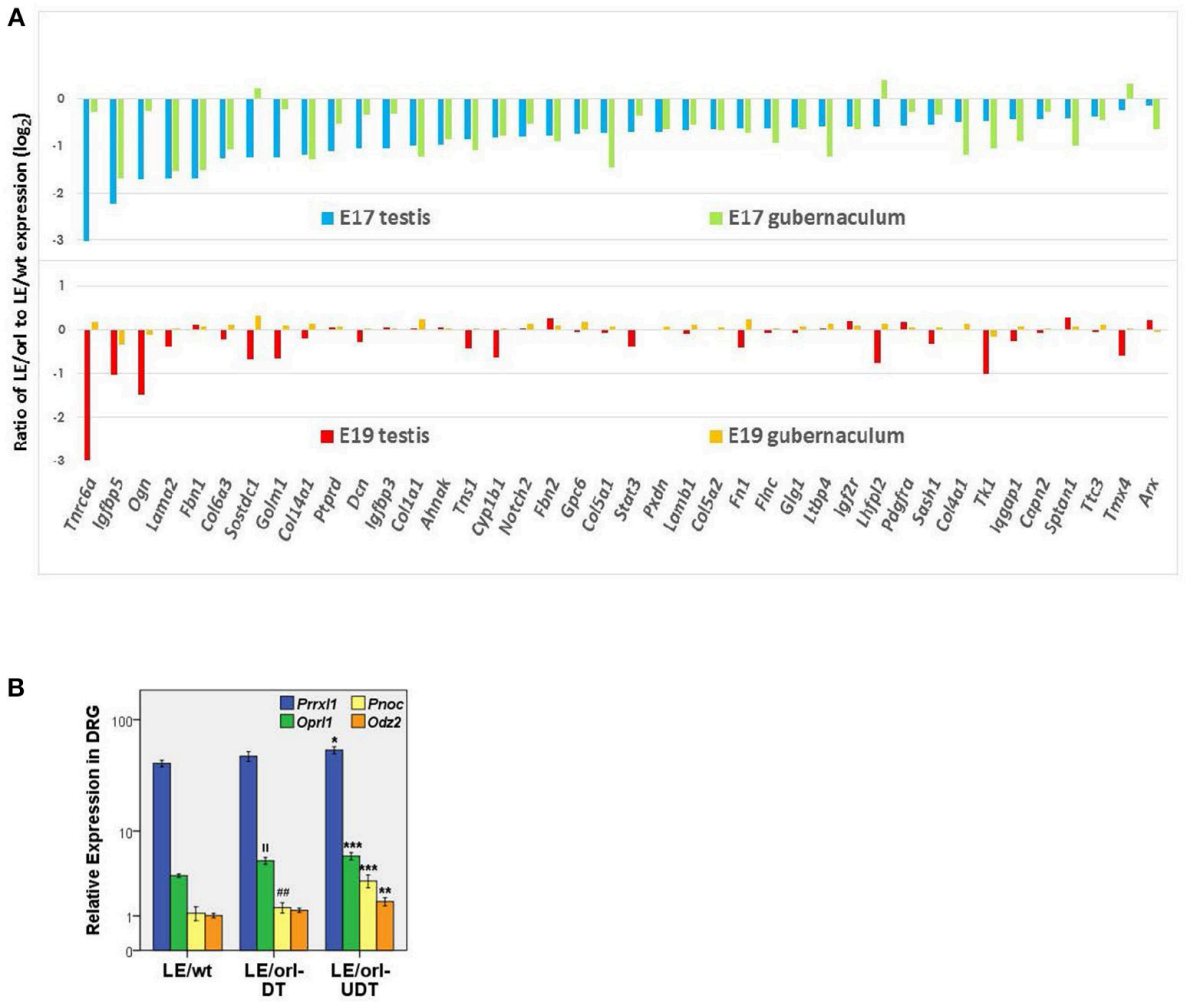
**(A)** Of 315 differentially expressed genes in LE/orl testis (45), 62 (20%) are Leydig cell progenitor-specific (46) and expression was reduced in 59 (95%) of these at E17. Forty of 62 (shown here) are also differentially expressed in LE/orl gubernaculum, and 37 of these (92%) are also downregulated. By E19, coordinate expression of these genes in these tissues is lost. **(B)** Expression levels of the sensory transcripts *Prrxl1* (paired related homeobox protein-like 1, also known as *Drg11* or *Drgx*), *Pnoc* (prepronociceptin) and its receptor, *Oprl1* (opioid receptor-like 1), and *Odz2* (odd oz/ten-m homolog 2, also known as *Tenm2*) measured by qRT-PCR (logarithmic mean ± SEM) in LE/wt and LE/orl L1-L2 dorsal root ganglia (DRG) ipsilateral to descended (DT) or undescended (UDT) testes. ***p < 0.001, **p < 0.01, *p < 0.05 vs. LE/wt; ^||^*p* < 0.01 vs. LE/wt; ^##^*p* < 0.01 vs. LE/orl-UDT by ANOVA; *n* = 5–10 per group, postnatal day 3 DRGs.

The Long Evans-derived LE/orl rat exhibits incompletely penetrant cryptorchidism that is associated with variants in at least 2 genes, *Syne2* and *Ncoa4*, which encode AR-interacting proteins (ARIPs) (38, 39). As frequently observed in cryptorchid rats exposed prenatally to anti-androgens (40–42) and cryptorchid boys, affected LE/orl testes are located in the superficial inguinal pouch, suggesting that this strain is a good model for a common form of clinical cryptorchidism. Testosterone levels and DHT-responsive transcript expression are reduced in LE/orl males, suggesting altered AR signaling (43). Testosterone deficiency alone is likely not sufficient to cause cryptorchidism in this strain, since other work suggests that a more marked reduction in Leydig cell hormone production is required to elicit this effect (44). Interestingly, in a recent re-analysis of transcriptome data (45) based on new information (46), we found that 110 of 315 (35%) differentially expressed LE/orl fetal testis transcripts map to genes whose expression is enriched in Leydig cell progenitors (*n* = 62; *p* = 2 × 10^−24^) or fetal Leydig cells (*n* = 48; *p* = 4 × 10^−11^; Fisher's exact test using Ingenuity Pathway Analysis/IPA®). The majority (59 of 62, 95%) of Leydig cell progenitor-enriched genes are downregulated at E17 in LE/orl as compared to the parent outbred strain (LE/wt). In addition, 40 of these transcripts are also differentially expressed in fetal gubernaculum, of which 37 (92%) are similarly downregulated. This evident coordinate gene regulation is lost by E19 (Figure 1A). These data are consistent with work published by the Agoulnik lab, which has shown that a retinoic acid receptor β type 2 Cre transgene is expressed in both Leydig cell progenitors and gubernaculum, and that conditional deletion of *Ar* in these cells impairs both testicular descent and fetal Leydig cell survival (17, 47). Others have confirmed that Leydig cell progenitors express AR (48), raising the possibility that the mesenchymal progenitors in the testicular interstitium and the gubernaculum may have a common origin, making AR important for both testicular hormone production and response.

LE/orl rats also carry a homozygous insertion within the *Prrxl1* (*Drg11, Drgx)* gene that is inherited together with the *Ncoa4* variant. *Prrxl1* regulates development of sensory neural circuitry (49) and transgenic deletion in mice leads to loss of CGRP-expressing neurons through apoptosis (50). Using qRT-PCR as described previously (51), we found that *Prrxl1* and other sensory transcript levels are altered in the L1-L2 dorsal root ganglia (DRG) of postnatal LE/orl rats, particularly ipsilateral to cryptorchid testes (Figure 1B). These data suggest that altered development of an integrated system comprising Leydig cells, sensory nerves and the gubernaculum together augment the risk of cryptorchidism in LE/orl rat fetuses. Yet even with apparent defects at multiple levels, at least half of all LE/orl testes descend normally. Moreover, we must be cautious when dealing with the potential complexities of gene-environment interaction, and the anatomical and contextual differences between humans and other mammals. The levels of endocrine disrupting chemicals (EDC) required to cause cryptorchidism in experimental animals are much higher than the typical range of human exposures; yet genetic heterogeneity and the combined effects of multiple environmental influences may put some boys at increased risk. The complexity of the spontaneous LE/orl rat model of cryptorchidism may provide insight into the complexity of cryptorchidism in humans.

## The etiology of human cryptorchidism is complex, and likely associated with genetic and environmental factors

Subtle Leydig cell dysfunction, characterized by increased variance in INSL3 levels (52, 53) and hence increased risk for susceptibility to other factors, and reduced testosterone/luteinizing hormone (T/LH) ratio (54–56), may occur in boys with cryptorchidism. It is unclear if these defects are primary or secondary, genetic or environmental. Our genome-wide association study (GWAS) of cryptorchidism identified many suggestive signals, but none surpassed the genome-wide significance threshold (57–59), typical of a polygenic disorder. Pathway analysis of suggestive intragenic signals showed enrichment of genes encoding proteins involved in cytoskeletal functions, including known or predicted ARIPs. Thus, complementary human and rat data suggest that cryptorchidism susceptibility is heterogeneous, multilocus and potentially multifactorial.

## RBFOX proteins may function as major regulators of neuro-hormonal signaling in testicular descent and contribute to cryptorchidism susceptibility

**Figure 2:**
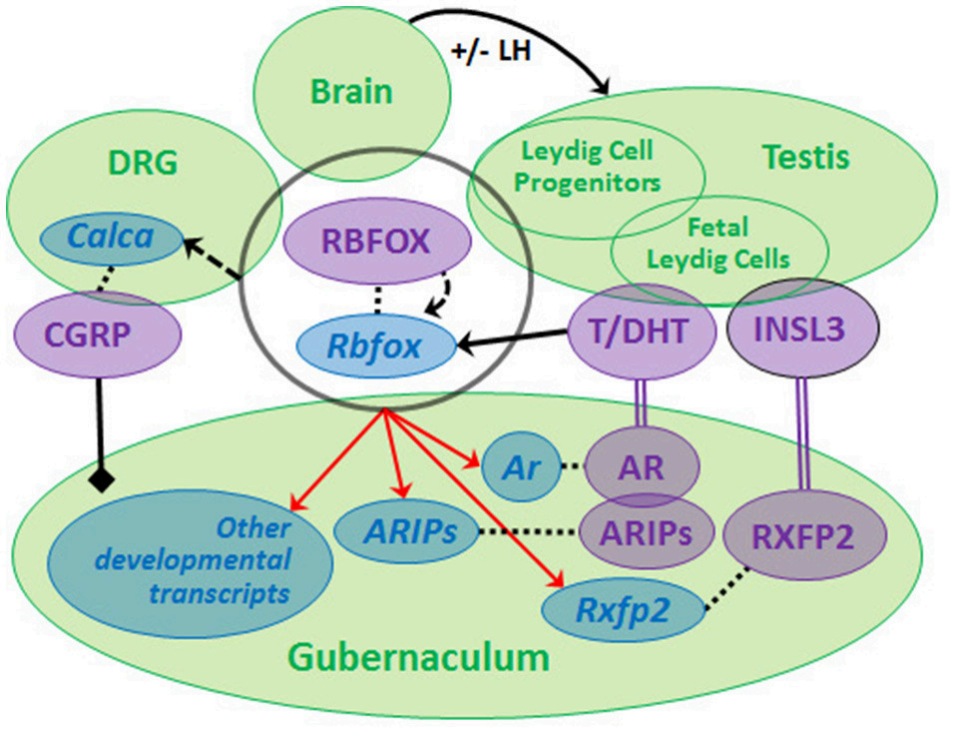
We propose that a neuro-hormonal signaling network regulates testicular descent via direct and indirect interactions among multiple target tissues (green circles). Development of the gubernaculum requires androgen (T, DHT) and insulin-like 3 (INSL3) binding, respectively, to RXFP2 and AR (together with AR-interacting proteins, ARIPs), and regulation by calcitonin gene-related peptide (CGRP). LH regulates Leydig cells during later phases of gestation. Rbfox/RBFOX transcripts/proteins are expressed (overlapping black circle) at all levels of the system with evidence for autoregulation (curved dashed arrow) and for regulation (solid straight arrow) by androgens [data from (46, 60), Barthold et al., unpublished]. RBFOXs regulate alternative splicing of *Calca* (dashed straight arrow) in dorsal root ganglia (DRG) to generate CGRP, which is released by sensory nerves innervating the gubernaculum (solid black line/diamond). *Ar, Rxfp2* and other developmental transcripts are predicted experimental and/or computational RBFOX targets (as denoted by red arrows; http://lulab.life.tsinghua.edu.cn/postar/). Androgens and INSL3 (20, 21) and possibly CGRP regulate other developmental transcripts in the fetal gubernaculum (not shown). Transcripts (blue italic) and proteins (purple, capital letters) are linked by dotted lines.

Recently, we compared GWAS data from non-syndromic cryptorchidism cases vs. controls (57) and from men with TGCT with or without a history of cryptorchidism vs. controls, and discovered suggestive signals in 19 genes, including *RBFOX1* and *RBFOX3*, paralogs that encode RNA-binding proteins (RBPs) (60). We found that predicted RBFOX targets are strongly enriched among developmental or differentially expressed Leydig cell- and gubernaculum-specific transcripts. The RBFOX proteins have relevant functions that include sex determination (61) and alternative splicing of *Calca* to produce CGRP (62). *Rbfox1* and *Rbfox2* are expressed in the rat fetal gubernaculum and L1-L2 DRGs, which produce the CGRP needed for gubernacular development, and *Rbfox2* expression is increased by DHT (data not shown). Based on these observations, we hypothesize that a neuro-hormonal RBFOX-AR-INSL3-CGRP signaling network regulating testicular descent may exist (Figure 2). We base this model on the neuro-hormonal data from rodent models, and these novel human genetic data suggesting a role for RBFOX genes in cryptorchidism susceptibility. Together, the human and rat data suggest that RBFOX family genes expressed in gubernaculum, testis and DRG (Figure 2) may regulate themselves and each other, playing complex roles in post-transcriptional regulation of CGRP, hormone receptor and/or other developmental molecules. The RBFOX family may therefore connect the hormonal and neural components of this complex network. Genetic variation impacting this network may interact (locally and/or systemically) with adverse effects of environmental endocrine disrupting chemicals (EDCs), augmenting susceptibility to cryptorchidism.

## Concluding synthesis

A feature of cryptorchidism which we need to take into account is that its etiology is primarily occurring in the fetus at a time shortly after sex determination when hormonal regulation is largely via local diffusion-based processes, and not by systemic circulation-borne events (52). This probably accounts for the preponderance of unilateral, as opposed to bilateral cryptorchidism and the prevalence of ipsilateral rather than general associations between factors. Localized regulatory networks such as we describe here, which may become differentially susceptible through increased variance (statistical “noise”) to a range of environmental or possibly epigenetic effects, are unlikely to reveal causality in single elements (genes or hormones) especially when using insensitive and systemic methodological approaches. Moreover, such localized and complex networks are linked to a highly dynamic and irreversible pathway of events, making them increasingly prone to stochastic/serendipitous localized influences, or dosage effects.

The complexity of such pathways (Figure 2) could explain the general failure to identify specific genes or EDCs associated with clinical cryptorchidism. Such data inform our perspective and underscore the need for a broader approach, utilizing systems biology and predictive modeling, to increase the likelihood of identifying both the causes and consequences of cryptorchidism.

## Ethics statement

This study was carried out in accordance in a facility accredited by the Association for Assessment and Accreditation of Laboratory Animal Care International. The protocol was approved by the Nemours Animal Care and Use Committee (ACUC).

## Author contributions

JB collected and analyzed original data, and JB and RI wrote the manuscript.

### Conflict of interest statement

The authors declare that the research was conducted in the absence of any commercial or financial relationships that could be construed as a potential conflict of interest.
